# Microbiota diversity and gene expression dynamics in human oral biofilms

**DOI:** 10.1186/1471-2164-15-311

**Published:** 2014-04-27

**Authors:** Alfonso Benítez-Páez, Pedro Belda-Ferre, Aurea Simón-Soro, Alex Mira

**Affiliations:** 1 Oral Microbiome Group – Department of Health and Genomics, Center for Advanced Research in Public Health (CSISP-FISABIO), Avda. Catalunya 21, 46020 Valencia, Spain; 2 Bioinformatics Analysis Group – GABi. Centro de Investigación y Desarrollo en Biotecnología (CIDBIO), Bogotá, D.C 111221, Colombia

**Keywords:** Dental plaque, Metatranscriptomics, Biofilm formation, Human microbiome, RT-qPCR, RNAseq

## Abstract

**Background:**

Micro-organisms inhabiting teeth surfaces grow on biofilms where a specific and complex succession of bacteria has been described by co-aggregation tests and DNA-based studies. Although the composition of oral biofilms is well established, the active portion of the bacterial community and the patterns of gene expression *in vivo* have not been studied.

**Results:**

Using RNA-sequencing technologies, we present the first metatranscriptomic study of human dental plaque, performed by two different approaches: (1) A short-reads, high-coverage approach by Illumina sequencing to characterize the gene activity repertoire of the microbial community during biofilm development; (2) A long-reads, lower-coverage approach by pyrosequencing to determine the taxonomic identity of the active microbiome before and after a meal ingestion. The high-coverage approach allowed us to analyze over 398 million reads, revealing that microbial communities are individual-specific and no bacterial species was detected as key player at any time during biofilm formation. We could identify some gene expression patterns characteristic for early and mature oral biofilms. The transcriptomic profile of several adhesion genes was confirmed through qPCR by measuring expression of fimbriae-associated genes. In addition to the specific set of gene functions overexpressed in early and mature oral biofilms, as detected through the short-reads dataset, the long-reads approach detected specific changes when comparing the metatranscriptome of the same individual before and after a meal, which can narrow down the list of organisms responsible for acid production and therefore potentially involved in dental caries.

**Conclusions:**

The bacteria changing activity during biofilm formation and after meal ingestion were person-specific. Interestingly, some individuals showed extreme homeostasis with virtually no changes in the active bacterial population after food ingestion, suggesting the presence of a microbial community which could be associated to dental health.

## Background

The study of microbial communities from environment- and human-derived samples through Next Generation Sequencing (NGS) methods has revealed a vast complexity in those ecological niches where hundreds or thousands of microbial species co-inhabit and functionally interact. One of these complex communities is that found in the human oral dental plaque (hereinafter, human oral biofilm). Although some studies, using NGS methods and 16S rRNA-based analysis, estimate that microbial diversity of the oral cavity is composed by thousands of species [1], more recent data have limited these estimates to a few hundreds [2-4]. Contrary to Koch's postulates, dental caries is not considered etiologically the outcome of a single-agent but is associated to an unbalance of microbial species that synergistically cause enamel demineralization by their acidogenic activity [5,6]. Thus, characterizing the composition of whole bacterial communities that actively engage in biofilm formation and sugar fermentation after the ingestion of food is vital for understanding community dynamics under health and disease conditions [7].

Although the set of species present in the human oral biofilm is almost fully depicted, new efforts have to be conducted to establish microbial agonistic or antagonistic associations, to distinguish actively-growing bacteria from inactive or transient species, as well as to outline the role of individual species during biofilm formation on tooth surfaces. The co-aggregation detected to occur between streptococci and *Actinomyces* species has been proposed to be a major promoter of human oral biofilm formation [8]. Like most biofilms, the dental plaque is built in a continued process characterized by succession of different bacterial species, each one with relevant roles in every step of biofilm construction [9]. Formation of the oral biofilm could be dissected in three major stages, namely: i) attachment; ii) colonization; and iii) biofilm development [10]. However, species participating of the entire process are traditionally characterized as “early” and “late” colonizers, where early colonizers would be responsible of the two first stages [9]. Among early colonizers the viridans streptococci group is considered as a cornerstone of the oral biofilm puzzle given its ability to bind saliva proteins through Antigens I and II. In this manner, streptococci species become the first colonizers able to bind tooth surfaces and promoting arrival of secondary colonizers by intergeneric coaggregation (reviewed in [9]). *Actinomyces naeslundii* is one of the secondary colonizers and a well known coaggregation partner of streptococci [8,11]. *Fusobacterium nucleatum* is considered a key player given its capability to coaggregate both with early and late colonizers of the oral biofilm [12], the latter group characterized by species belonging to Bacteroidetes and Spirochaetes [6,9]. It is noteworthy to highlight that intergeneric coaggregation not only contributes to bacterial growth and colonization [13], but it is thought to facilitate the genetic and metabolic exchange among species, and even to create the adequate environment for arrival of some obligate anaerobic bacteria [10]. Therefore, any disruption in the development of the oral biofilm caused by impairing of early colonizers tooth attachment or inability to recruit other key players during biofilm formation, would affect the entire process avoiding presence of pathogens responsible for periodontal disease or caries [7].

Although few attempts to link specific gene expression profiles in oral bacteria with the establishment and maturation of oral biofilm have been done [14], further studies are needed to understand global gene dynamics and intracellular signalling which are the basis for cell-to-cell communication among oral bacteria and to promote biofilm formation on tooth surfaces. There are important limitations to study gene expression from *in vivo* oral samples, including RNA instability and amounts of sampling material, but a sequencing approach of total cDNA from an *in vitro* oral biofilm model has recently been performed [15].

Because gene transcripts typically occupy a small fraction of total bacterial RNA, even after mRNA-enriching protocols, a massive coverage is normally required to quantify gene expression by RNAseq technologies. On the other hand, high-coverage sequencing technologies are normally coupled to short read lengths, which jeopardize accurate taxonomic assignment of the sequences. The latter can be achieved through the use of longer reads, at the expense of a lower coverage of mRNA transcripts. In the present manuscript we present the first metatranscriptome analysis of *in vivo* human oral biofilm samples through two approaches: A short read-length, high coverage Illumina® approach to study oral biofilm formation through time, and a long read-length, lower coverage pyrosequencing strategy to study changes in community composition before and after a meal. For the first approach, a total of 16 samples of supragingival plaque from 4 healthy individuals were collected at four different time points (6, 12, 24 and 48 hours after a professional ultrasound cleaning) to disclose the microbiota and gene expression dynamics during oral biofilm formation. For the second experiment, the metatranscriptome of dental plaque from five individuals was studied 30 minutes before and after a controlled meal, in order to characterize the potential shifts in the active bacterial community when dietary nutrients are available for growth.

## Results & discussion

### Metagenome vs metatranscriptome

**Figure 1:**
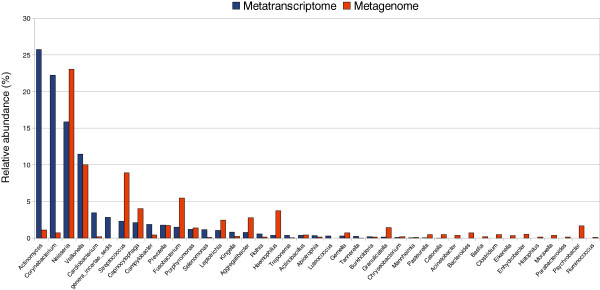
**Total (DNA-based) and active (RNA-based) microbiota composition in the human oral biofilm from individual NoCa-01.** Microbial diversity is inferred from 16S rDNA and 16S cDNA taxonomic assignment, respectively, from reads obtained by direct pyrosequencing of the DNA and cDNA of a 24 h dental plaque sample. Relative abundance of bacterial genera from metagenomic data differs from that obtained from metatranscriptomic data. The former would correspond to the total bacterial composition in the sample whereas the latter would represent the “active microbiota” as inferred by their presence in samples’ RNA.

A preliminary test was performed by direct pyrosequencing of DNA (sequence data obtained from [3]) and cDNA from a 24-hour dental plaque sample from the same individual (NoCa1). The results show a very different pattern of bacterial genera in the metagenome and the metatranscriptome (Figure 1). *Actinomyces*, *Corynebacterium* and *Neisseria* were the three most abundant genera in the RNA-based community whereas *Veillonella*, *Streptococcus* and *Leptotrichia* were the most commonly found in the total DNA-based metagenome. In addition, a long tail of low-proportion genera is observed in the metagenome but absent in the metatranscriptome, suggesting they could correspond to transient or inactive bacteria. This shows the importance of obtaining both kind of data to understand the composition and dynamics of human-associated microbial populations.

### Low-coverage, long-reads approach

#### Active communities before and after a meal

**Figure 2:**
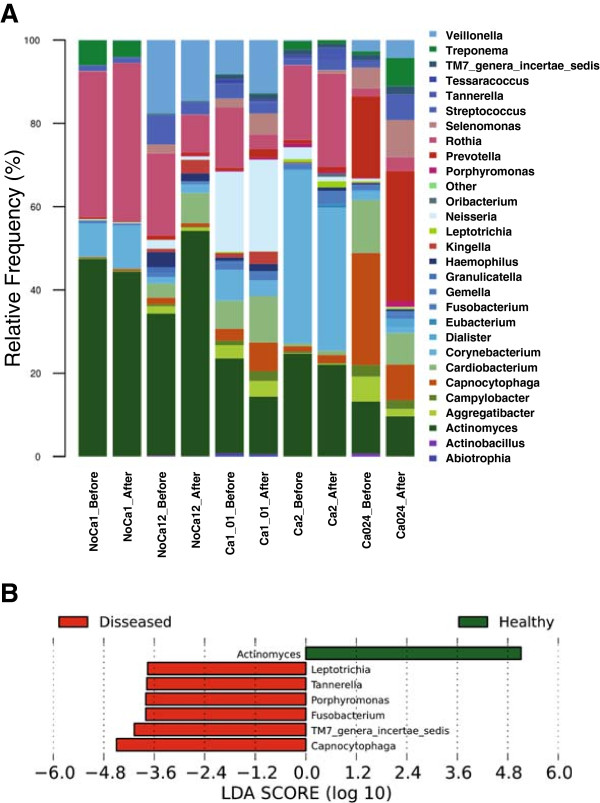
**Meal-uptake-dependent active microbiota and association with health and disease. A** – Graphical representation of the genera distribution according to 16S rRNA assignation of metatranscriptomic reads (obtained by pyrosequencng of total cDNA) based on RDP classifier. Relative frequency for most predominant active genera is shown in dental plaque samples obtained before and after a carbohydrate-rich meal. **B** – Health- and disease-associated genera in the above metatranscriptome, as inferred from Linear Discrimination Analysis (LDA) performed for dimensional class comparisons. The data were generated by using LEfSe test, available in the Galaxy Web Server toolkit. NoCa = individuals with no caries; Ca = individuals with caries.

A total of 213,419 pyrosequencing reads were obtained after quality filtering [16-18]. An average of 38.9% corresponded to SSU rRNA sequences, 59.9% to LSU rRNA and 1.2% to other sequences, including mRNA. Taxonomic assignments based on 16S and 23S rDNA sequences gave similar results (Figure 2A and Additional file 1: Figure S1). A different bacterial composition was found for each individual. In some cases, over 80% of active bacteria corresponded to only three genera (for instance *Actinomyces*, *Corynebacterium* and *Rothia* for individuals NoCa1 and Ca2) whereas other individuals did not show any dominant genera in their active microbial community (Figure 2A, Additional file 2: Table S3). Some individuals were very resilient to changes after the meal (e.g. individual NoCa1), whereas others had more apparent changes in the proportions of some bacteria, but no specific pattern was common to all individuals (Additional file 3: Figure S2). Thus, the changes in active bacteria after a meal were not universal and depended on the original microbial population associated to each human host.

*Actinomyces* was the only genus found at a proportion over 10% in all samples and was found to be significantly more abundant in healthy individuals (Figure 2B) according to a high-dimensional class comparison test [19]. *Actinomyces* is an early colonizer of the oral biofilm, usually present in the first layers of dental plaque in contact with enamel [11]. It is able to increase local pH by producing ammonia through the degradation of arginine, lysilarginine and urea [20]. As a consequence, individual *Actinomyces* species could represent biomarkers of healthy biofilms with a protective role against acidogenic bacteria [21]. On the other hand, late colonizers being strictly anaerobes like *Porphyromonas*, *Fusobacterium*, *Capnocytophaga*, *Tannerella* and *Leptotrichia* were found significantly more abundant in oral biofilm from caries-bearing individuals (Figure 2B). This should be further studied given the observed over-representation of species belonging to the red complex of periodontal disease [22].

Although the number of assigned mRNA transcripts detected by this approach is quite small, we could identify some genes being expressed in all samples such as those encoding ribosomal proteins and basic housekeeping machinery like elongation factors and cell division proteins. Interestingly, we found expression of multiple sugar transport systems and central metabolism genes such as glyceraldehyde 3-phosphate dehydrogenase, L-lactate dehydrogenase, citrate synthase, enolase, and malate dehydrogenase.

### High-coverage, short-reads approach

#### Microbial activity during oral biofilm formation

Although the high-coverage approach (~25 million Illumina reads per sample) was mainly aimed at determining gene expression patterns during biofilm formation, an attempt was first made to utilize the reads mapping to rRNA genes to characterize microbiota composition at different times of dental plaque formation. Potential errors in taxonomic assignment were minimized by 1) assigning reads at the family and genus taxonomic level only; 2) selecting matches against 16S rRNA database of 100% sequence identity; and 3) eliminating hits to conserved regions of the 16S rRNA gene, keeping only hypervariable, informative regions.

**Figure 3:**
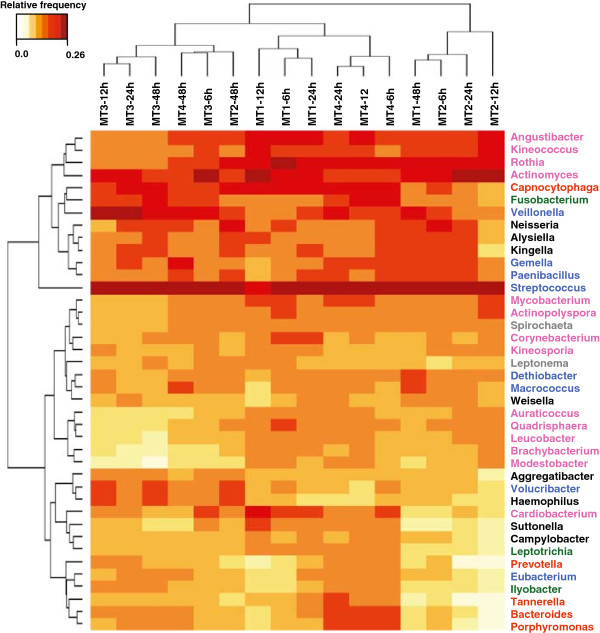
**Clustering of oral biofilm samples according to the estimated microbial diversity.** Log2 transformed frequencies of top 40 genera found in the dental plaque metatranscriptome through time are shown. Metatranscriptome samples were identified with MT prefix followed by time point (6, 12, 24, or 48 hours of biofilm formation). Number after dot refers to donor code. Bacterial genera labels are highlighted in colour according to phylum: Actinobacteria (pink), Bacteroidetes (Orange), Fusobacteria (Green), Firmicutes (Blue), Proteobacteria (Black), and Spirochaetes (Grey).

When we tried to compare samples according to maturation time we found that samples from early biofilm (6 and 12 h) showed significantly (*p* ≤ 0.0118) less genera than samples from mature biofilm (24 and 48 h). In average, 171 genera were found at a frequency above 0.01%, accounting for 99.4% of the global diversity. The 40 most predominant genera found in all analyzed samples are depicted in Figure 3. These 40 genera account for 68% of gene expression according to their frequency in the metatranscriptomic reads. The heat-map in Figure 3 shows the genus-level clustering according to frequency within each sample. Among predominant genera we could observe *Streptococcus* (found at relative abundances between 12 to 19% in different samples) and *Actinomyces* (in a range of 3-12%), both being well known partners for coaggregation [8,9]. Interestingly, *Actinomyces* showed higher frequencies in early biofilm samples, in agreement with its known role as early colonizer. In addition to *Streptococcus* and *Actinomyces,* other frequent genera were the Actinobacteria *Rothia, Angustibacter,* and *Kineococcus*; the Proteobacteria *Neisseria*, *Kingella* and *Alysiella*; the Firmicutes *Gemella, Paenibacillus* and *Veillonella,* the latter also reported as coaggregation partner with *Streptococcus*[23]; and finally *Capnocytophaga* and *Fusobacterium.* When we tried to discern a specific pattern of microbial organisms associated with different times of biofilm formation it was observed that samples predominantly clustered according to the donor they were extracted. Consequently, we could detect no clear association between bacterial composition and biofilm development stage. These results would globally fit within the concept that individual-specific microbial communities are a consequence of host-bacterial co-evolution to maintain host health [24,25]. Consequently, the host-specific microbiota could be considered as a genetic fingerprint almost unique for every person ([25,26] and references therein), and even preserved throughout the years in a very stable fashion [27].

#### Microbial interactions during oral biofilm formation

**Figure 4:**
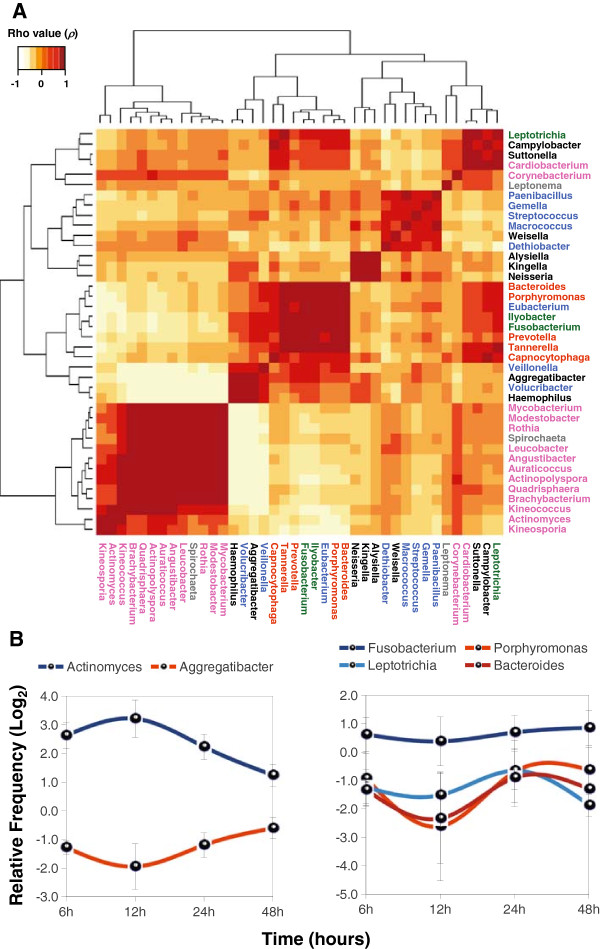
**Positive and negative interactions in the oral biofilm active microbiota. A** – Relative abundance of bacterial genera (based on 16S assignment, see Methods) and fluctuation through time were studied in a pairwise manner calculating the Spearman’s rho parameter (see Methods). Genera labels are highlighted in color according to phylum: Actinobacteria (pink), Bacteroidetes (Orange), Fusobacteria (Green), Firmicutes (Blue), Proteobacteria (Black), and Spirochaetes (Grey). **B** – Detail of the Spearman correlations found in the analysis. The left panel shows a strong negative (*ρ* ~ - 0.75) correlation between *Actinomyces* and *Aggregatibacter* through time. In the right panel, a positive correlations is presented among late colonizers such as *Leptotrichia*, *Fusobacterium*, *Porphyromonas,* and *Bacteroides* (*ρ* ~ 0.80).

Although no association was found between specific bacteria and biofilm stage, we observed certain correlation patterns between different microbial groups which were reproducible in different patients. In order to detect monotonic functions associated to genera frequency fluctuation through time, we calculated the Spearman's rho parameter (*ρ*) for the top 40 more predominant genera listed in Figure 3 for all patients. Thus, we could obtain a map of significant positive and negative correlations that can indicate either pairwise interactions between genera or adaptation to similar environmental conditions (Figure 4A). Interestingly, most genera belonging to the same phylum showed positive correlations. In this way, Actinobacteria members (*ρ* = 0.7346 in average) appeared to show the same growth pattern during biofilm formation as well as Fusobacteria and Bacteroidetes (*ρ* = 0.7833 and 0.7450 on average, respectively). In contrast, genera assigned to Proteobacteria and Firmicutes showed lower correlation values (*ρ* < 0.34) because some species within these groups had different patterns of occurrence. Globally, several genera seem to have a negative correlation with Actinobacteria, particularly *Veillonella* (Firmicutes) *Volucribacter* (Proteobacteria), *Haemophilus* (Proteobacteria)*,* and *Aggregatibacter* (Proteobacteria)*,* the latter showing strong negative correlations against 11 out of 15 different genera of Actinobacteria detected (Figure 4A). An example is given in Figure 4B (left panel), which shows the distribution of *Actinomyces* versus *Aggregatibacter* (*ρ* = -0.7581) during biofilm evolution. A quasi-mirror distribution is observed between these two genera suggesting that arrival and/or growth of *Actinomyces* could be outcompeted by *Aggregatibacter* presence in biofilm. In contrast, a multiple positive correlation is exemplified by the distribution of *Fusobacterium, Bacteroides, Porphyromonas* and *Leptotrichia* (Figure 4B, right panel) in full agreement with the coaggregation partners established for *Fusobacterium* and the classical view of species succession during oral biofilm formation and the establishment of late colonizers [9,12]. Indeed, Fusobacteria species seem to have the same distribution pattern than Bacteroidetes given the multiple significant positive correlations observed among their genera (Figure 4A). The presence of potential periodontal pathogens [28] in these multiple correlation patterns could be indicative of their synergy for arrival to the oral biofilm and probably for development of periodontal disease.

#### In vivo gene expression and functional analysis of the oral biofilm

For the functional analysis of gene expression patterns during oral biofilm formation the KEGG functional classification [29] was used. We detected expression of 19,519 genes (ORFs with aligned reads) on average per sample and the set of KO categories represented was 2,266, indicating that ~ 12% of expressed genes were functionally annotated. A distinguishable clustering according to biofilm stage was not fully depicted, although a pairwise clustering among early and mature biofilm samples was observed (data not shown). Some molecular pathways were differentially expressed between early and mature biofilm (i.e. Ribosome; Purine Metabolism; and Glycolysis). A comparison was performed between early (6 h and 12 h) and mature (24 h and 48 h) biofilm samples, determining the False Discovery Rate (FDR) with q-value ≤ 0.05. We detected a set of 271 KO categories differentially expressed (35 over-expressed in early biofilm and 236 in mature biofilm).

#### Over-expression in early biofilm

Over-expression of KO categories in early biofilm was predominantly grouped in genes involved in the metabolism of Carbohydrates, Energy, Amino Acids, Cofactor/Vitamins, and Xenobiotic Degradation. Translation functions were also overrepresented because several ribosome proteins showed higher expression during early biofilm as well as the Elongation Factors Tu (EF-Tu, K02358) and G (EF-G, K02355). Central role of Translation in early steps of oral biofilm formation was also evidenced by over-expression of K00566 category corresponding to the MnmA tRNA-modifying protein. MnmA is an evolutionarily conserved enzyme and incorporates the posttranscriptional modification s^2^U at the wobble position of several tRNAs [30] which reads A/G ending codons during translation. The tRNA modifications are largely associated to control the fine-tuning of protein synthesis, thus improving ribosome accuracy [31]. As a consequence, they appear to be involved in controlling a wide range of bacterial phenotypic traits including biofilm formation by multi-drug resistant human pathogens [32] and could be important for oral biofilm formation as well. Our results are in agreement with previous reports where amino acid metabolism is critical for growth of early colonizers such as *Streptococcus gordonii,* which needs coaggregation to stabilize expression of genes involved in amino acid synthesis and membrane transporters [14].

#### Over-expression in mature biofilm

On the other hand, genes over-expressed at the late biofilm stage had a more variable functional profile. New functional categories over-expressed in late oral biofilms included the ABC transporters, the Cell motility represented by the orthology groups K03407 associated to bacterial chemotaxis, K02676 and K02390 involved in pilus and flagella assembly, respectively, and finally genes involved in Base Excision Repair, Mismatch Repair, and Homologous Recombination systems; and some putative transposases. The group of tRNA-modifying enzymes was also present in late oral biofilm with a larger set of genes over-expressed such as *mnmC*, *yfiC, cmoA, tadA, queE*, *trmK*, and the hydrouridine synthase gene *dusC.* Consequently, this molecular pathway seems to be involved in controlling the expression of proteins along the full oral biofilm process. Strikingly, a set of genes involved in competition between bacterial species also showed over-expression during this late stage of oral biofilm formation. The *comFC, comFA, comGB, comGC,* and *comGA* orthology groups are members of the Type II secretion system of which other members appear to be annotated as Competence-related DNA transformation transporters. These genes have been reported to be involved in quorum sensing response to produce mutacins; these are non-lantibiotic bacteriocins able to induce lysis and consequently DNA liberation in related species possibly supporting DNA horizontal transfer [33]. Likewise, *tfoX* orthologues were also found to be over-expressed in late oral biofilm, thus strengthening the idea of natural genetic transformation [34,35] occurring among close species in the mature oral biofilm. Globally, over-expression of these competence-related genes, permitting DNA transformations *in vivo,* could support the specific low ratio between functional diversity of genes and operational taxonomic units detected in supragingival plaque, thus indicating high functional redundancy and microbial population homogenization [36]. Other important functional categories over-expressed in late oral biofilm included those involved in Environmental Information Processing and membrane transporters, such as those belonging to the Phosphotransferase System (PTS) as well as MFS membrane receptors specialized in the importing/exporting of small molecules. Both major families of membrane transporters were found to be preferentially expressed from *Actinomyces* species indicating a high level of metabolic exchange between this genus and its environment. However, subfamilies such a *salX*-like ABC transporters associated to bacteriocin export and defense were detected to be predominantly active in streptococci species. Over-expression of some KEGG orthology categories belonging to Two-Component family of proteins indicate an active role of cells in perceiving external signals of nutrient availability in the environment. An over-expression was found of the PTS-Ntr-EIIA enzyme and the GlnB protein, both involved in nitrogen regulation, and the sigma factor 54 of the RNA polymerase involved in expression of genes for nitrogen metabolism. Therefore, processes related to nitrogen uptake/metabolism appear to be very relevant in the mature stage of oral biofilm probably indicating that nitrogen is a limiting factor for oral biofilm progression. In recent studies of cDNA massive sequencing from an *in vitro* five-species oral biofilm microbial community, similar results were obtained in terms of overrepresented functions in mature biofilms [15]. Finally, *luxS* homologue in *Neisseria* spp. was found to be significantly over-expressed in early biofilm. The *luxS* genes are responsible of Autoinducer-2 (AI-2) synthesis, a molecule considered as a major interspecies signal for cell-cell communication [9,10]. Evidence for AI-2 role to control biofilm formation was previously observed when a *luxS* null strain of *S. gordonii* was unable to form a mixed-species biofilm with *P. gingivalis*[37].

#### Gene expression by qPCR

**Figure 5:**
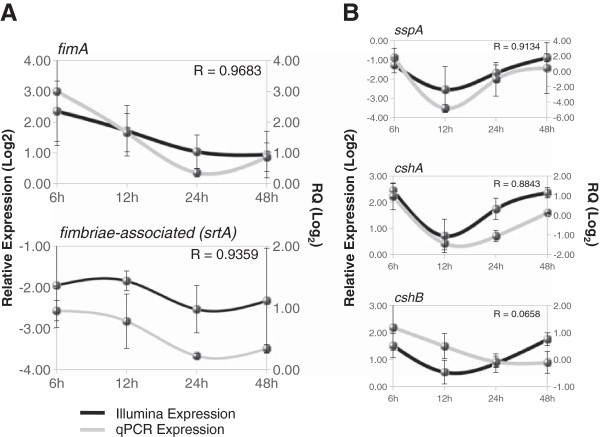
**Gene expression comparison between Illumina sequencing and qPCR during biofilm formation. A** - Genes associated to Type 2 Fimbriae assembly in *Actinomyces naeslundii* were analyzed and Pearson correlations calculated from expression values obtained by these two approaches. **B** – Adhesion genes from *Streptococcus gordonii* were also analyzed and their expression pattern was compared.

We selected adhesion genes involved in cell-to-matrix and cell-to-cell interactions to corroborate by qPCR the expression pattern inferred from Illumina sequencing. Among the adhesins we could find several molecules such as SspA and SspB proteins from *S. gordonii* and homologues present in several species of *Streptococcus,* all being very relevant for attachment to tooth surfaces [9]. Type 1 and Type 2 Fimbriae molecules found in *Actinomyces* species are described to mediate coaggregation with streptococci species. The surface expression of before mentioned proteins is sortase A (SrtA) dependent [9,38,39], catalyzing a peptidic linking to the cell wall [40] and promoting interactions with the extracellular molecules from bacterial counterparts or host tissues in the case of pathogens [39]. Regarding the role of *Actinomyces* sp*.* Type 2 Fimbriae in coaggregation with *Streptococcus* sp*.*[8,41]*,* we studied the expression pattern of its gene (*fimA*) together with the *A. naeslundii* Fimbriae-Associated protein gene (*srtA*) and some other adhesins from *Streptococcus gordonii*. The Illumina- and qPCR-derived expression patterns during the oral biofilm formation for *fimA* and *srtA* homologue from *A. naeslundii* are showed in Figure 5A. qPCR data showed that these two genes had similar expression patterns, thus suggesting a co-expression pattern and quite probably dependent on their clustered localization in the *Actinomyces naeslundii* chromosome. We found that expression patterns were similar at all time points of oral biofilm formation from all patients with high correlation coefficients. In addition to the high degree of correlation between Illumina and qPCR expression data, all expression patterns of these genes present a common feature, namely a high level of expression at very early stage of oral biofilm formation and then decaying in a slight or noticeable manner. Once we showed gene expression dynamics of adhesins from *A. naeslundii* and *S. gordonii* during oral biofilm formation, we observed our data is in agreement with other *in vivo* analysis performed by immunodetection of surface molecules by fluorescence labelling and co-localization [8]. SspA protein is involved in attachment of *Streptococcus gordonii* to the tooth surface by recognizing salivary agglutinins and it also mediates interaction with *Actinomyces* sp*.*[42,43]. The expression profile from *sspA* from *S. gordonii* is presented in Figure 5B (top panel). The correlation coefficient between qPCR and Illumina data is ~ 0.91. Its expression pattern is similar to that found for Type 2 Fimbriae genes from *A. naeslundii*, which is in agreement with the role of streptococci as first colonizers and the requirement of SspA for attaching to the tooth surface and promote arrival of other early colonizers such as *Actinomyces* sp*.*[9,44]. Finally, we studied the expression patterns of *cshA* and *cshB* genes coding for two cell surface antigens in *S. gordonii* that increase hydrophobicity of cell surface and mediate interactions with *A. naeslundii* and human fibronectin [45,46]. Expression profiles for *cshA* and *cshB* obtained by qPCR (Figure 5B) are fairly similar to that observed for *sspA* gene and correlation with Illumina sequencing data is notable, at least for *cshA*. Expression patterns of different adhesion proteins analyzed showed a similar pattern, with higher expression in very early stages of oral biofilm. In addition, most of the adhesion genes studied here showed an increased level of expression at the end point of study (48 h). We hypothesize that such level of expression would reflect the last stage of the biofilm cycle where biofilm detachment occurs, thus releasing bacterial cells to colonize new niches [10,47].

## Conclusions

Our study shows for the first time the microbial diversity and gene expression dynamics in the complex oral microbial community *in vivo*. We could follow oral biofilm formation and determine proportions of active microbiota through time, including before and after a carbohydrate-rich meal, when the process of acid production, responsible of enamel demineralization, takes place. We present a large set of correlations among bacterial groups and genera being in agreement with biological and classical interactions reported to be central for biofilm installation and development [8,48,49]. In the functional exploration of genes expressed during human oral biofilm formation, we present a quantitative analysis, further supported by results obtained by qPCR, demonstrating several functional categories of prevalence at different oral biofilm stages. Among them we showed that translation machinery is predominantly expressed in early biofilm stages whereas more specialized genes are required in mature biofilm. Some genes involved in competence, and reported to be involved in quorum sensing response and functionally related to mutacin production and DNA uptake, were over-expressed in late biofilm supporting the intricate level of cell-to-cell interactions in mature biofilm and suggesting strong competition for colonization. More than 70% of the genetic information compiled from this oral metatranscriptome has no functional assignment; therefore, further efforts must be conducted for classification and characterization of genes and their involvement in biofilm development and/or cell-to-cell communication. From an applied point of view, the identification of active bacterial species after food uptake can be considered a first step to narrow down the list of potential etiological agents of dental caries from the large set of micro-organisms found in the metagenome of dental plaque and cavities [3]. The striking homeostasis found in one of the individuals who had never suffered from dental caries, and where virtually no changes were found in the active microbiota before and after a meal, could indicate that the microbiota of some individuals is not affected by food ingestion, potentially reducing the risk of acidic pH and promoting dental health.

## Methods

### Sample collection and RNA processing

The sampling procedure was approved by the Ethical Committee for Clinical Research from the DGSP-CSISP (Valencian Health Authority, Spain) and all donors signed an informed consent. The oral health status of each individual was evaluated before sampling and following recommendations and nomenclature from the WHO. Donors were 20-30 years of age, had all 28 teeth present (excluding third molars) had not suffered from any systemic disease and had not taken systemic antimicrobials in the previous 6 months. Dental plaque samples were taken with autoclaved spoon excavators from vestibular and lingual surfaces of teeth excluding a 1 mm region on the edges.

For the biofilm formation experiments, 16 supragingival dental plaque samples were obtained from 4 caries-free volunteers (DMFT = 0 [decayed, Missing, Filling Teeth], OHI = 1 [oral Hygiene Index]. GI = 1 [gingival Index]). The volunteers were subjected to professional teeth ultrasound cleaning. Oral biofilm (supragingival plaque) from all teeth surfaces was pooled and collected from each volunteer at 6, 12, 24, and 48 h of biofilm formation. After every sampling a professional brushing was performed to reset biofilm formation for next sampling. Total RNA was extracted using the MasterPure^TM^ RNA Purification Kit (Epicentre®). Samples were collected and processed for elimination of 5S rRNA and tRNAs through ion exchange chromatography with KCl gradient in Nucleobond AX 20 columns (Macherey-Nagel). Pre- and post-processed RNA were loaded in RNA chip (Agilent Technologies) and analyzed for integrity using Agilent Bioanalyzer 2100 (Agilent Technologies). The first strand of cDNA from processed RNA was synthesized using High-Capacity cDNA Reverse Transcription Kit (Applied Biosystems). For this aim, two cDNA reactions were prepared for each RNA sample and modifying some manufacturer’s instructions to obtain best performance during synthesis. Specifically, each cDNA reaction had 100U of Multiscribe Reverse transcriptase and synthesis was completed at 48°C during 210 min using 5-10 ug of RNA as template. Doubled stranded cDNA (ds-cDNA) was achieved in 100 uL of reaction containing 35U *E.coli* DNA Polymerase I (New England Biolabs), 5U *E. coli* DNA Ligase (New England Biolabs), 5U RNase H (Epicentre®), 300 uM dNTP's, and two reactions of first strand cDNA synthesis. The ds-cDNA synthesis was initiated by incubation during 150 min at 16°C and completed by adding 4.5U of T4 DNA Polymerase (New England Biolabs), and 1X BSA (New England Biolabs) followed by incubation during additional 30 min at 16°C. Purified ds-cDNAs were obtained using High Pure PCR Product Purification Kit (Roche®) and sent to GATC Biotech AG (Konstanz, Germany) for parallel single-end sequencing using HiSeq2000 system (Illumina®).

The five donors for the before/after meal transcriptome were asked not to brush their teeth for 16 hours. Three of them had active caries at the moment of sampling (Decayed Teeth = 3, OHI = 1, GI = 1) and the other two had no history of dental caries (DMFT = 0, OHI = 1, GI = 1). None of the donors had periodontal disease. All donors ingested the same meal, whose nutritional characteristics are indicated in Additional file 4: Table S1. Supragingival dental plaque was obtained from the right maxillary and left mandibular quadrant free teeth surfaces for the sample 30 minutes before eating and from the left maxillary and right mandibular quadrant 30 minutes after food intake, without touching caries lesions, if they were present. Opposite quadrants were sampled because, when analysing PCR-amplified 16S rRNA from cDNA, an equivalence in terms of taxonomic composition was found when sampling opposite mandibular and maxillary quadrants (Additional file 5: Figure S3). The obtained total ds-cDNA (as described above) was purified and enriched in fragments longer than 400 bp using AMPure beads (Agencourt). Those long cDNA fragments were sequenced using 454 GS-FLX technology with titanium chemistry (Roche).

### Taxonomic assignment and correlations

Filtering and trimming of original data set was assisted by Galaxy Web Server [16-18], filtering by quality using the sliding window method (window size 25 with a minimum quality in the window of 20), and sequences shorter than 200 bp were removed. For the high-coverage biofilm samples, microbial diversity was established by taxonomic assignment using reads matching 16S rRNA sequences. For this aim we constructed a RDP-based (Release 10, Update 29) database containing almost 10,000 reference sequences of 16S rDNA annotated according to NCBI taxonomy [50]. This reference database was processed to filter out the conserved regions of 16S rDNA genes using Hidden Markov Models [51]. Then, using MegaBlast v2.2.21 algorithm [52] and selecting alignments for 48 nt in length and 100% identity we could assign taxonomy at genus level using only hypervariable regions of 16S rDNA sequences, thus determining predominant microbiota. Heat maps of taxonomic composition were generated using the gplots library of R [53], frequencies were log_2_ transformed and clustered with Euclidean distance. In the case of samples before/after a meal, microbial diversity was established using the 16S and 23S rRNA gene. 16S and 23S sequences were binned using META-RNA 1.0 [54]. 16S sequences were assigned using the online RDP assigner [50]. 23S sequences were assigned using the SILVA database and SINA assigner [55,56]. All statistical analyses were conducted on R v2.15. Non-parametric Spearman rank correlation was calculated among top 40 most frequent genera to associate frequency fluctuations during biofilm formation between genera. Then, Spearman's (*ρ*) coefficient and *t*-test significance was calculated for pairs of genera from all patients, using a Bonferroni correction for multiple comparisons.

### Functional analysis

Based on predominant microbiota present at all states of biofilm formation, available complete and WGS genomes were retrieved from the RefSeq and the Human Oral Microbiome databases [57,58]. More than 80 genomes of oral related microorganisms were downloaded and used to build a local database with almost 300,000 coding sequences. More than 800 small predicted ORFs (100-400 nt) were removed, being 98-100% identical to different regions of 16S or 23S rDNAs [59]. The remaining set of ORFs were then submitted to the KEGG Automatic Annotation Server [29] for KEGG Orthology (KO) assignment. Using MegaBlast v2.2.21 algorithm [52] with e-value cutoff 1e-08 and selecting alignments longer than 60% of read with >80% of identity, we assigned KO numbers and PATH categories to the BRITE functional hierarchy [29]. Negative binomial distribution contained in DESeq [60] bioconductor v2.10 package (default parameters) was employed for differential expression analysis. KO over-representation was determined by comparison between early (6-12 h) and late (24-48 h) biofilm samples with q values ≤ 0.05. Counting of reads per gene and genome were normalized against genus frequency and size dataset and then transformed in log_2_ for comparison with qPCR expression data.

### Quantitative PCR

Primers for qPCR were designed submitting the respective ORF sequences from *S. gordonii* and *A. naeslundii* to the Primer3Plus webserver [61] (Additional file 6: Table S2). Gene amplification was performed using LightCycler® 480 System (Roche), SYBR Green I Master (Roche), and a small aliquot from the respective sample sequenced by Illumina. The Cp values were calculated from three replicates using the LightCycler® 480 SW software v1.5 (Roche). Expression was normalized against 16S rRNA expression from *S. gordonii* and *A. naeslundii*, respectively, and referred to expression seen for every gene at 6h for all patients in average using the ΔΔCt method.

### Data access

All sequence data derived from 454 pyrosequencing of cDNA from samples after/before meal experiments, microbial diversity associated to dental quadrants, and Illumina HiSeq2000 sequencing of cDNA from oral biofilm are stored in the MG-RAST server to be publicly available by accessing to the “Oral Metatranscriptome” project, id 935 (http://metagenomics.anl.gov/linkin.cgi?project=935). Sequence data is also available at the European Nucleotide Archive (ENA-EBML) with provisional accession number ERP003984.

## Competing interests

The authors declare that they have no competing interests.

## Authors’ contributions

All authors participated in the study design. ASS carried out the sampling. ABP and PBF performed the molecular biology studies and high-throughput data analysis. ABP, PBF and AM worked in manuscript preparation. All authors read and approved the final manuscript.

## Supplementary Material

Additional file 1: Figure S1 Bacterial genera composition according to 23S rDNA. The taxonomic assignation was based on SINA analysis against reference samples from the SILVA database. Bars show the relative frequency for most predominant genera in metatranscriptomic samples obtained before and after a carbohydrate-rich meal. Click here for file

Additional file 2: Table S3 Shannon Diversity Indexes for samples from the low-coverage approach. Click here for file

Additional file 3: Figure S2 Bacterial relative abundances between samples obtained before and after a meal. Positive values (expressed as log_2_ ratios) are colored in green and indicate a higher abundance of a given genus in the sample before the meal; negative values (also expressed as log_2_ ratios), colored in red, indicate a higher abundance in the after-meal sample. Click here for file

Additional file 4: Table S1 Number of reads analyzed for taxonomy assignment from the low-coverage approach. Click here for file

Additional file 5: Figure S3 Bacterial diversity analysis of the 24 h human oral biofilm according to dental quadrants. Bacterial composition was estimated by pyrosequencing of the 16S rRNA gene obtained by PCR amplification of cDNA. Diversity at the family taxonomic level (Actinobacteria as Phylum) was determined in biofilm samples coming from four dental quadrants of a unique donor. Pie charts for every quadrant show relative frequency for most predominant bacterial families. Rarefaction curves for each quadrant display a similar diversity for all samples and bacterial composition piecharts indicate slight differences at the frequency of some families like Neisseriaceae being less frequent in upper quadrants. Click here for file

Additional file 6: Table S2 Sequence information for oligonucleotides used in the qPCR approach. Click here for file
