# Supplementary figures and images for: Microbiota diversity and gene expression dynamics in human oral biofilms
Source: BMC Genomics. 2014 Apr 27;15:311. doi: 10.1186/1471-2164-15-311 (PMC4234424; doi:10.1186/1471-2164-15-311)

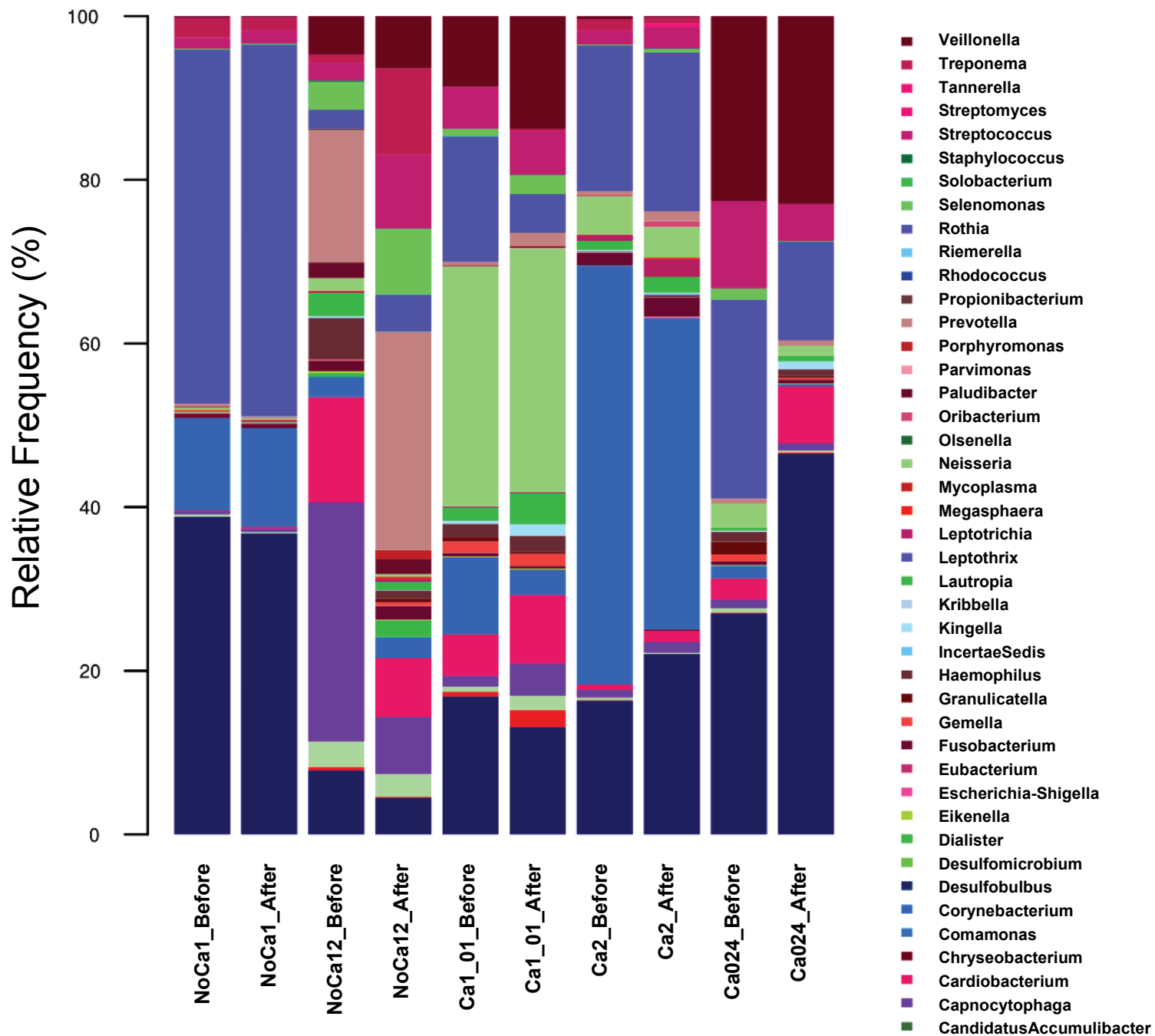

Supplement: Additional file 1: Figure S1 — Bacterial genera composition according to 23S rDNA. The taxonomic assignation was based on SINA analysis against reference samples from the SILVA database. Bars show the relative frequency for most predominant genera in metatranscriptomic samples obtained before and after a carbohydrate-rich meal. [file 1471-2164-15-311-S1.pdf]

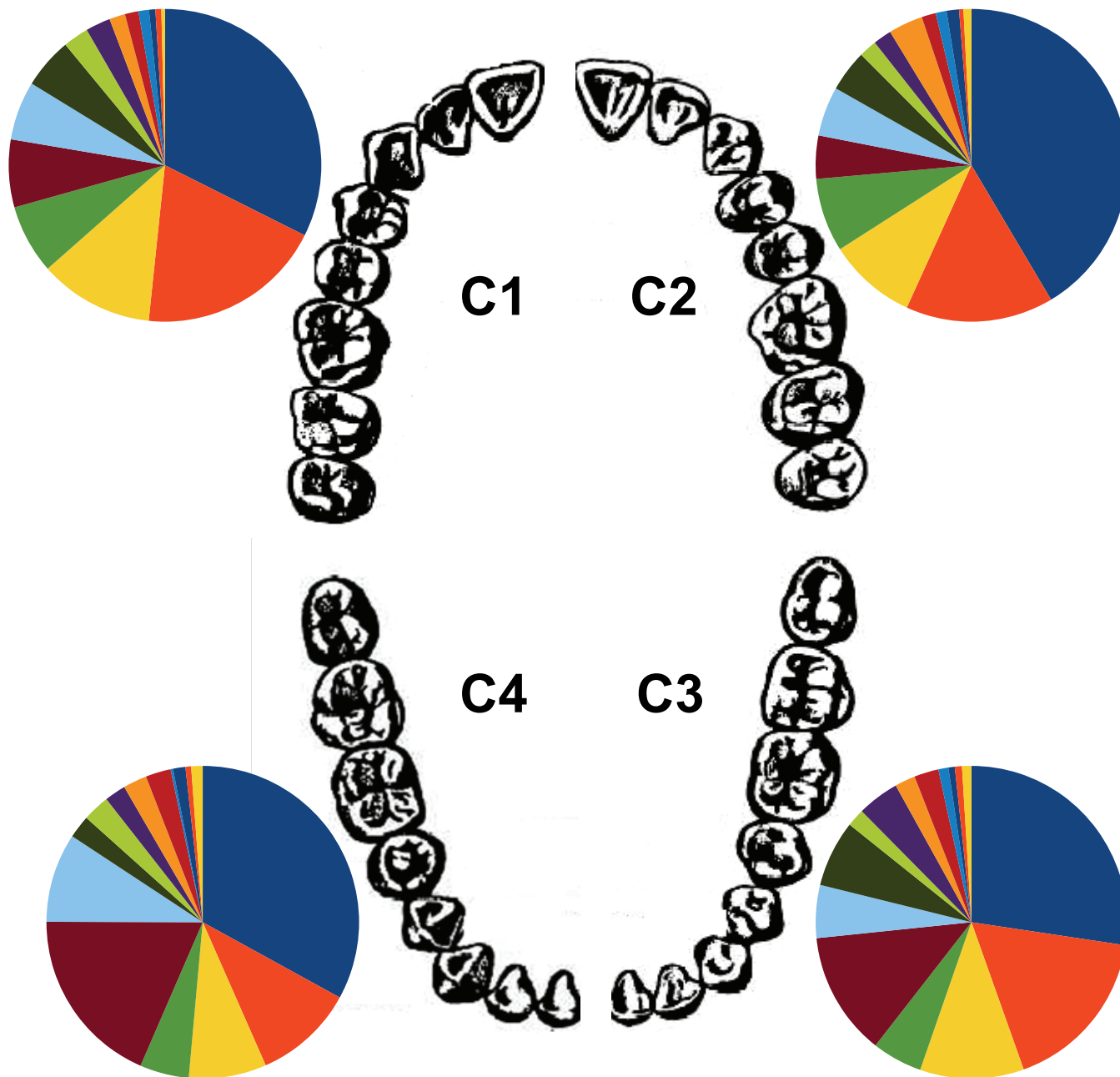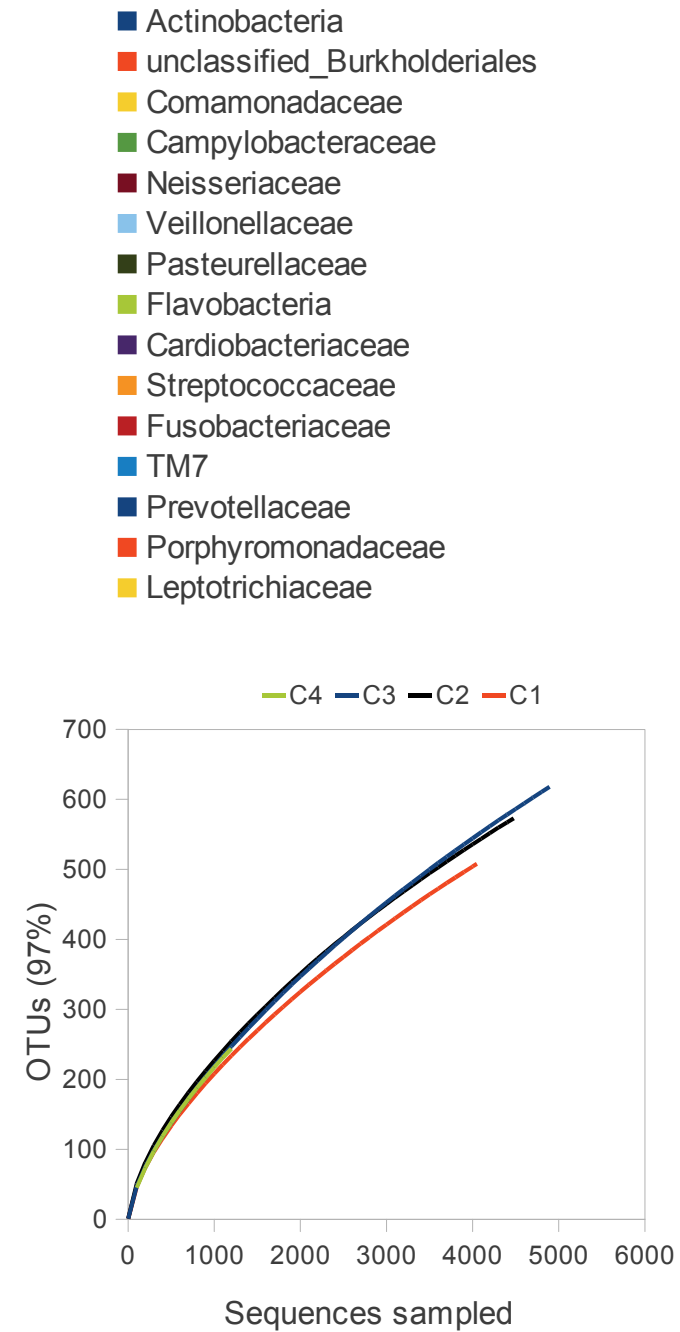

Supplement: Additional file 5: Figure S3 — Bacterial diversity analysis of the 24 h human oral biofilm according to dental quadrants. Bacterial composition was estimated by pyrosequencing of the 16S rRNA gene obtained by PCR amplification of cDNA. Diversity at the family taxonomic level (Actinobacteria as Phylum) was determined in biofilm samples coming from four dental quadrants of a unique donor. Pie charts for every quadrant show relative frequency for most predominant bacterial families. Rarefaction curves for each quadrant display a similar diversity for all samples and bacterial composition piecharts indicate slight differences at the frequency of some families like Neisseriaceae being less frequent in upper quadrants. [file 1471-2164-15-311-S5.pdf]
