# Supplementary material for: Microbiota diversity and gene expression dynamics in human oral biofilms
Source: BMC Genomics. 2014 Apr 27;15:311. doi: 10.1186/1471-2164-15-311 (PMC4234424; doi:10.1186/1471-2164-15-311)
Supplement: Additional file 2: Table S3 — Shannon Diversity Indexes for samples from the low-coverage approach. [file 1471-2164-15-311-S2.doc]

**Table S3. Shannon Diversity Indexes for samples from the low-coverage approach.**

| **Sample** | **Shannon-Wiever index** |
| --- | --- |
| NoCa1_Before | 1,2930 |
| NoCa1_After | 1,2982 |
| Ca024_Before | 2,2537 |
| Ca024_After | 2,4085 |
| Ca1_01_Before | 2,3883 |
| Ca1_01_After | 2,5276 |
| Ca2_Before | 1,7289 |
| Ca2_After | 1,9196 |
| NoCa12_Before | 2,0443 |
| NoCa12_After | 1,6381 |
