# Supplementary material for: Microbiota diversity and gene expression dynamics in human oral biofilms
Source: BMC Genomics. 2014 Apr 27;15:311. doi: 10.1186/1471-2164-15-311 (PMC4234424; doi:10.1186/1471-2164-15-311)
Supplement: Additional file 3: Figure S2 — Bacterial relative abundances between samples obtained before and after a meal. Positive values (expressed as log2 ratios) are colored in green and indicate a higher abundance of a given genus in the sample before the meal; negative values (also expressed as log2 ratios), colored in red, indicate a higher abundance in the after-meal sample. [file 1471-2164-15-311-S3.pdf]

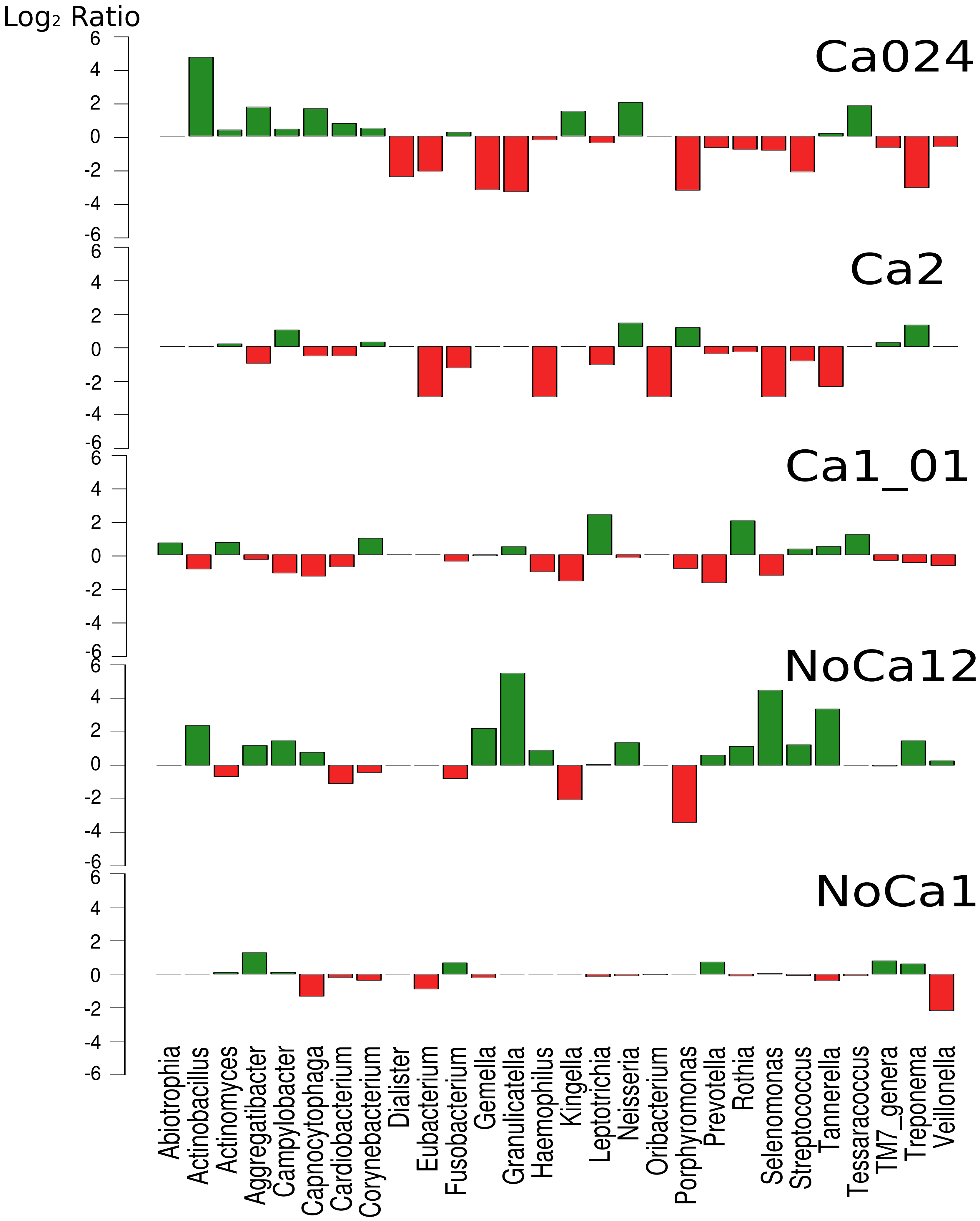

Log2 Ratio
Ca024
Ca2
Ca1_01
NoCa12
NoCa1
6
4
2
0
-2
-4
-6
Abiotrophia
Actinobacillus
Actinomyces
Aggregatibacter
Campylobacter
Capnocytophaga
Cardiobacterium
Corynebacterium
Dialister
Eubacterium
Fusobacterium
Gemella
Granulicatella
Haemophilus
Kingella
Leptotrichia
Neisseria
Oribacterium
Porphyromonas
Prevotella
Rothia
Selenomonas
Streptococcus
Tannerella
Tessaracoccus
TM7_genera
Treponema
Veillonella
