# Supplementary material for: Microbiota diversity and gene expression dynamics in human oral biofilms
Source: BMC Genomics. 2014 Apr 27;15:311. doi: 10.1186/1471-2164-15-311 (PMC4234424; doi:10.1186/1471-2164-15-311)
Supplement: Additional file 4: Table S1 — Number of reads analyzed for taxonomy assignment from the low-coverage approach. [file 1471-2164-15-311-S4.doc]

**Table S1. Number of reads analyzed for taxonomy assignment from the low-coverage approach.**

| Sequence number | 16S | 23S | Other (mRNA) | Total sequences |
| --- | --- | --- | --- | --- |
| NoCa1 Before | 9769 | 12706 | 596 | 23071 |
| NoCa1 After | 23497 | 34589 | 1348 | 59434 |
| NoCa12 Before | 6626 | 11576 | 112 | 18314 |
| NoCa12 After | 6779 | 10117 | 179 | 17075 |
| Ca1_01 Before | 16500 | 26825 | 386 | 43711 |
| Ca1_01 After | 6695 | 9479 | 181 | 16355 |
| Ca2 Before | 5501 | 9238 | 186 | 14925 |
| Ca2 After | 561 | 1051 | 10 | 1622 |
| Ca024 Before | 2539 | 3647 | 66 | 6252 |
| Ca024 After | 5092 | 7398 | 170 | 12660 |
