# Supplementary material for: Microbiota diversity and gene expression dynamics in human oral biofilms
Source: BMC Genomics. 2014 Apr 27;15:311. doi: 10.1186/1471-2164-15-311 (PMC4234424; doi:10.1186/1471-2164-15-311)
Supplement: Additional file 6: Table S2 — Sequence information for oligonucleotides used in the qPCR approach. [file 1471-2164-15-311-S6.doc]

**Table S2. Sequence information for oligonucleotides used in the qPCR approach.**

| **Gene**  **(species)** | **Name** | **Sequence** | **Gene Position** | **Tm (Cº)** | **Amplicon Size (bp)** |
| --- | --- | --- | --- | --- | --- |
| *cshA* | CshA-F | TTC CAT TCC CAG CTG ATT CGA CT | 4361 | 62.9 | 100 |
| (*S. gordonii*) | CshA-R | ACC TTA CCG TCT GCG TCC AC | 4460 | 62.5 | 100 |
| *cshB* | CshB-F | TCC GGC TAG CTT TGT GGA TGC | 2487 | 63.2 | 116 |
| (*S.gordonii*) | CshB-R | TCA CTT GGC CGG TAT TTG GAT C | 2602 | 62.2 | 116 |
| *fimA* | FimA-F | GAC GGC CAG TGG ATC TAC GA | 556 | 62.5 | 126 |
| (*A. naeslundii*) | FimA-R | GCT CAC CGG GAA CTT GAT GAG | 681 | 63.2 | 126 |
| *srtA* | Fimbriae-F | CGT CGA GGT CTT CGG AGA GG | 477 | 64.6 | 114 |
| (*A. naeslundii*) | Fimbriae-R | ACC AGG GTG AGC AGG TCC TT | 590 | 62.5 | 114 |
| *sspA* | SspA-F | CTT GGT ATG GTG CAG GGG CTA | 2261 | 63.2 | 103 |
| (*S. gordonii*) | SspA-R | TGA GGC ATT TCC GCT ACA GGC | 2363 | 63.2 | 103 |
| *sspB* | SspB-F | CGA CCG GAC ATT GGT TGC TAA AC | 2811 | 64.6 | 118 |
| (*S. gordonii*) | SspB-R | GCC AGT TGG AAG CGG ATC TAC | 2928 | 63.2 | 118 |
| 16S rRNA | 16S_strp-F | GGG GAT AAC TAT TGG AAA CGA TAG C | 147 | 64.1 | 115 |
| (*S. gordonii*) | 16S_strp-R | ACT AGC TAA TAC AAC GCA GGT CCA T | 261 | 64.1 | 115 |
| 16S rRNA | 16S_acti-F | GAG TAA CAC GTG AGT AAC CTG CC | 99 | 64.6 | 150 |
| (*A. naeslundii*) | 16S_acti-R | GAT AGG CCG CGA GCC CAT C | 248 | 63.6 | 150 |
